# Construction of Functional Materials in Various Material Forms from Cellulosic Cholesteric Liquid Crystals

**DOI:** 10.3390/nano11112969

**Published:** 2021-11-05

**Authors:** Kazuma Miyagi, Yoshikuni Teramoto

**Affiliations:** 1 Department of Forest Resource Chemistry, Forestry and Forest Products Research Institute, Forest Research and Management Organization, 1 Matsunosato, Tsukuba 3058687, Ibaraki, Japan; 2 Division of Forest and Biomaterials Science, Graduate School of Agriculture, Kyoto University, Kitashirakawa Oiwake-cho, Sakyo-ku, Kyoto 6068502, Japan

**Keywords:** cellulose derivatives, cellulose nanocrystal, cholesteric liquid crystal, functional materials, material form

## Abstract

Wide use of bio-based polymers could play a key role in facilitating a more sustainable society because such polymers are renewable and ecofriendly. Cellulose is a representative bio-based polymer and has been used in various materials. To further expand the application of cellulose, it is crucial to develop functional materials utilizing cellulosic physicochemical properties that are acknowledged but insufficiently applied. Cellulose derivatives and cellulose nanocrystals exhibit a cholesteric liquid crystal (ChLC) property based on rigidity and chirality, and this property is promising for constructing next-generation functional materials. The form of such materials is an important factor because material form is closely related with function. To date, researchers have reported cellulosic ChLC materials with a wide range of material forms—such as films, gels, mesoporous materials, and emulsions—for diverse functions. We first briefly review the fundamental aspects of cellulosic ChLCs. Then we comprehensively review research on cellulosic ChLC functional materials in terms of their material forms. Thus, this review provides insights into the creation of novel cellulosic ChLC functional materials based on material form designed toward the expanded application of cellulosics.

## 1. General Introduction: Need for and Overview of Cellulosic Liquid-Crystals as Functional Materials

### 1.1. Introduction

Cellulose is the most abundant bioderived organic polymer. Because of its excellent physical properties, including its mechanical and sorption properties, cellulose is widely used in areas such as the manufacture of paper, textiles, and structural components. In addition to conventional utilization, developing applications of unused but attractive physicochemical properties of cellulose is important to realize the wealth of a sustainable society because cellulose is reproducible and eco-friendly.

Liquid crystallinity is one of the characteristic physical properties of cellulosic molecules, although it is not fully employed in the real world. The high stimuli-responsivity and high-order structure of liquid crystals may enable cellulosic functional materials. It is also interesting that the liquid crystal property develops spontaneously. Numerous fundamental studies on cellulosic liquid crystals have been reported. The creation of functional materials that take advantage of the insights gained in those studies may contribute to expanding the areas in which cellulosics can be used.

There are several excellent reviews on the fundamental science and possible applications of cellulosic liquid-crystals. For instance, Gray [1] summarized initial research on the liquid-crystallinity of several cellulose derivatives. Lagerwall et al. [2] recently reviewed the phase diagrams of aqueous cellulose nanocrystal (CNC) dispersions and discussed ordered assembly of CNCs in suspensions as well as films. The Lagerwall group has most recently published a significantly detailed review on equilibrium and kinetically arrested liquid crystalline structure based on CNC [3]. Giese et al. [4] reviewed cellulosic liquid crystalline structures in terms of their structural templates. Moreover, a review by Nishio et al. [5] surveyed both the fundamental aspects and potential applications of liquid crystalline cellulose derivatives and nanocrystals in terms of their stimuli responses. However, to our knowledge there is no review on cellulosic liquid crystalline functional materials in the context of material forms.

The material form is an important factor involving the function and application of materials. For example, films are applied for electronic and optical devices, hydrogels are compatible with biomedical applications, and porous materials are applied for water purification. In the present review, we therefore categorize previous studies on cellulosic liquid crystalline functional materials in broad terms to solid and liquid types, and comprehensively review various material forms corresponding to the respective types.

### 1.2. Cellulosic Liquid-Crystals

We here briefly describe the basic fundamentals of cellulosic liquid-crystals before considering their applications as functional materials.

**Figure 1:**
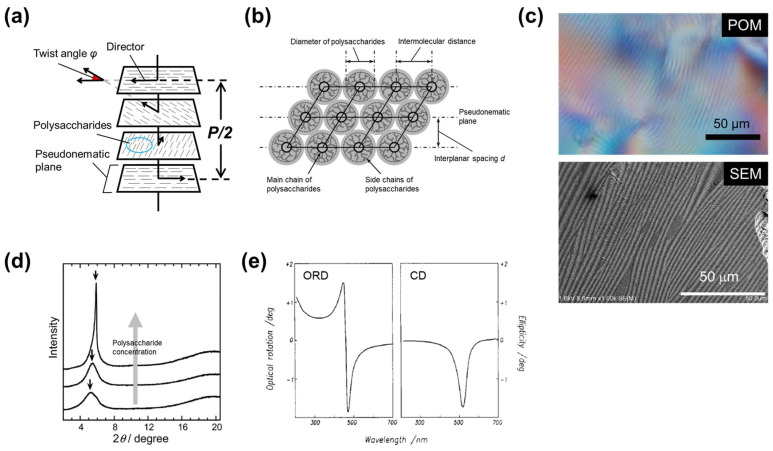
(**a**) Schematic that illustrates the helical pitch *P*, director, twist angle *φ* for a cholesteric liquid crystalline (ChLC) phase composed of polysaccharides. (**b**) Schematic illustrating the pseudonematic planes and interplanar spacing *d* for a short-range structure of a polysaccharide-based ChLC phase (reprinted with permission from [7] Copyright (2021) American Chemical Society). (**c**) Representative polarized optical microscope (POM) image of solution-state polysaccharide-based ChLC sample and scanning electron microscope (SEM) image of solid-state sample (reprinted with permission from [33] Copyright (2021) American Chemical Society). (**d**) Representative wide-angle X-ray diffraction profiles of solution-state polysaccharide-based ChLC samples with different polysaccharide concentrations (reprinted with permission from [7] Copyright (2021) American Chemical Society). (**e**) Representative circular dichroism (CD) and optical rotary dispersion (ORD) spectra of a right-handed ChLC sample (Reprinted with permission from [34] Copyright (2021) American Chemical Society).

Cellulose has a backbone chain consisting of β-1,4-linked glucose units that impart (semi-)rigidity and chirality. These structural features endow cellulosics with the potential to form a cholesteric liquid crystal (ChLC) phase. ChLC is often modeled as being formed from stacked virtual planes, each of which is composed of molecules or particles with a uniaxial orientation. The vector indicating such orientation is called director. The director of each plane (termed as pseudonematic plane) twists periodically around the normal of the stacked pseudonematic planes, rendering a helical nature to ChLC. Figure 1a presents a schematic illustration of ChLC with polysaccharides as component molecules. Although the schematic ChLC in Figure 1a seems to be a discretely layered structure, pseudonematic planes should be continuously stacked in actual ChLC. Figure 1b is a schematic diagram focusing on the short-range structure of a ChLC to emphasize such continuously stacked pseudonematic planes.

ChLCs can be attained for rigid and chiral particles or molecules in the dispersed or solution state (at a sufficiently high concentration) termed a lyotropic ChLC, or in a molten state termed a thermotropic ChLC. The classification of lyotropic and thermotropic states applies not only to ChLCs but also to liquid-crystals in general.

ChLCs exhibit a unique optical property: selective reflection of circularly polarized light (CPL) with a wavelength equivalent to the helical pitch and with a rotation direction that is the same as that of the helix. The reflection wavelength (*λ*_M_) and the helical pitch (*P*) are related by de Vries’ equation, *λ*_M_ = *ñP* (*ñ* is the refractive index) [6]. Accordingly, ChLCs exhibit circular dichroism (CD) as well as coloration, when *P* is comparable with a wavelength in the range of visible light. As stated in this equation, the cholesteric helical pitch *P* of ChLC samples can be calculated from their *λ*_M_ and *ñ* values, which can be acquired with a spectrophotometer and refractometer, respectively. The *P* value can also be estimated by microscopy; polarized optical microscope (POM) and scanning electron microscope (SEM) images of ChLC samples demonstrate the characteristic fingerprint-like texture (Figure 1c), and the distance between the adjacent dark lines corresponds to half of *P*. It should be noted that SEM can be used to estimate the *P* of solid-state ChLC samples, but not for solution-state (lyotropic) ones. Solidification of such solution samples by polymerization of their solvents described in Section 2.1 enables the measurement of *P* from an SEM image, although the solidification process can affect *P* to some extent. In accordance with the ChLC structure indicated in Figure 1a,b, *P* can be described as *P* = 360°*d*/*φ*, where *d* is the interplanar spacing of the stacked pseudonematic planes and *φ* is the twist angle in degrees. This indicates that *P* and thus *λ*_M_ change with *d* and *φ*. The value of *d* can be obtained from wide-angle X-ray diffraction analysis as shown in Figure 1d by approximating the short-range order of a ChLC with the hexagonal model [7]. The helical handedness of a ChLC is determined by CD or optical rotatory dispersion (ORD) spectroscopy. ChLC samples with a left-handed helix exhibit a positive peak in CD spectra and a positive Cotton effect in ORD spectra, whereas ChLC samples with a right-handed helix exhibit a negative peak in CD spectra and a negative Cotton effect in ORD spectra (Figure 1e). The helical handedness can also be discriminated by visual inspection of ChLC samples with circular polarizers, because left-handed and right-handed CPL (L-CPL and R-CPL, respectively) reflected from the samples can transmit through a left-handed circular polarizer (LCP) and a right-handed circular polarizer (RCP), respectively.

Expression of lyotropic ChLC requires rigid and chiral molecules or particles to have good solubility or dispersity in solvents, and thermotropic ChLC requires them to have good meltability. The native cellulose molecule has poor solubility and thermal meltability because of its strong inter- and intramolecular hydrogen bonding. Instead, cellulose derivatives and nanocrystals have typically been adopted to achieve a cellulosic ChLC phase.

The CNC is the structural unit of a cellulose crystal and can be produced by acid hydrolysis of cellulose microfibrils [8,9]. Cellulose microfibrils in wood are generally considered to be composed of bundles of 6 × 6 cellulose molecular chains, although there are recent reports on cellulose microfibrils comprising 18 molecular chains [10]. Such cellulose bundles exhibit alternating amorphous and crystalline regions along the molecular chain direction. Acid hydrolysis of cellulose bundles preferentially degrades the amorphous regions. After degrading most of the amorphous regions, the degradation is substantially decelerated, resulting in residual highly crystalline particles which are the CNCs. According to this characteristic, the degree of polymerization of cellulose chains in the crystalline regions is called the level-off degree of polymerization (the degradation “turns off” at this degree of polymerization) [11]. CNCs prepared by hydrolysis with sulfuric acid are an especially active area of research. CNCs can express lyotropic ChLC in aqueous suspensions based on CNCs’ good water-dispersity, which is attributable to the introduced surface sulfate ester groups [12,13]; whereas CNCs generally do not express thermotropic ChLCs because of CNCs’ lesser meltability. Since the first report of liquid-crystallinity in CNCs [14], fundamental research on CNC-based ChLCs have been made [15,16,17,18,19,20]. CNC-based ChLCs typically form a left-handed helix, and to our knowledge there has been no report on formation of a right-handed helix from CNCs to date.

Cellulose derivatives can be obtained by substituting the hydroxy groups in cellulose, typically by etherification or esterification [21]. Because substituents suppress the strong intra- and intermolecular hydrogen bonding of cellulose, cellulose derivatives often exhibit good solubility and meltability. Such properties facilitate expression of lyotropic and thermotropic ChLCs. The first reports of formation of ChLCs by cellulose derivatives were for an aqueous hydroxypropyl cellulose (HPC) solution system [22]. Since then, many important fundamental studies on cellulose derivative-based ChLCs have been presented—such as the effects of concentration, temperature, solvent, side chain structure, and degree of substitution on the selective reflection properties [23,24,25,26,27,28,29].

In contrast to CNCs, left- and right-handed cholesteric helices have been observed for cellulose derivative-based ChLCs. Most recently, Nishio et al. [30,31] proposed that the effects of the substituents and temperature on the helical handedness of cellulosic ChLCs can be systematically interpreted in terms of the balance between the dispersion interaction and steric repulsion, based on their experimental data and the theoretical work of Osipov [30,31].

There is a difference between the lyotropic ChLCs of cellulose derivatives and CNCs. For the former, the building blocks of the ChLC structure are molecules of cellulose derivatives, and the component molecules completely dissolve in solvents. Conversely, for the latter, CNC “particles” that disperse in solvents (rather than dissolve) provide the ChLC structure. Accordingly, ChLC systems comprising cellulose derivatives and CNCs are sometimes termed molecular ChLCs and colloidal ChLCs, respectively. The structural difference between cellulose derivatives and CNC leads to different characteristics of their ChLC samples. The representative examples include instances where the cholesteric helical pitch of cellulose derivative-based ChLCs is shorter than those of CNC’s with corresponding concentration because of the smaller diameter of cellulose derivatives (~1 nm) relative to CNC (5–20 nm [8,32]); and where the critical concentration to express lyotropic ChLC for CNC is lower than that of cellulose derivatives because of the very rigid crystal backbone of CNC.

Various insights accumulated by previous fundamental studies on cellulosic ChLCs have led to increased research activity dedicated to processing ChLCs into material forms intended for practical use. The present article reviews information on functional materials with diverse material forms produced from cellulose derivative-based and CNC-based ChLCs. The following review is divided into sections based on material forms, and we concurrently review both cellulose derivative-based and CNC-based functional materials. Please note that the difference in characteristics between the aforementioned ChLCs leads to different experimental conditions and procedures to construct the functional materials even in the same material forms.

## 2. Cellulosic ChLC-Based Solid Functional Materials

### 2.1. Cellulosic ChLC Films

Cellulose derivatives and CNC are useful components for producing films incorporating a ChLC structure. Representative methods for preparing cellulose derivative-based ChLC films are solution-casting [35,36,37] and in situ polymerization [38,39,40,41,42,43] of a lyotropic ChLC solution of cellulose derivatives. In solution-casting methods, typically the cellulosic ChLC solutions are first spread into a film-like shape and sandwiched (i.e., packed) between two glass plates, followed by slow evaporation of the solvents. Although this method is operationally simple, it insufficiently immobilizes ChLC structure with a long helical pitch *P* corresponding to a red wavelength because the cholesteric helical pitch decreases in accordance with increasing cellulosic concentration of the solutions over the course of drying. However, it is straightforward to immobilize a ChLC structure with a short *P* in considerably concentrated cellulosic solutions because of the extremely low mobility of the cellulosic chains. Regarding the in situ polymerization method, lyotropic ChLC solutions are prepared by dissolving cellulose derivatives in polymerizable solvents (e.g., acrylate and acrylamide monomers) and polymerization-initiator, and then the solution samples—molded between the glass plates—are subjected to heating or ultraviolet (UV) irradiation to initiate polymerization of the monomeric solvents. The ChLC structure formed in the solution state is retained even after the monomeric solvents are converted to polymeric matrices (e.g., polyacrylate and polyacrylamide), and these matrices help to further immobilize the ChLC structure. Thus, in situ polymerization methods can immobilize ChLC structures that have not only a short *P* but also a long *P*, unlike solution-casting methods. This renders in situ polymerization a general method for preparing cellulose derivative-based ChLC films. However, the ability to immobilize the ChLC structure strongly depends on compatibility between cellulose derivatives and the resultant synthetic polymers, because interaction of mesogenic cellulosic chains may be distorted when cellulose derivatives completely mix with the synthetic polymers, as described in Section 2.1.1.

For CNC-based ChLC films as well, solution (dispersion)-casting [2,12,44] and in situ polymerization [4,5] are representative preparation methods. In contrast to cellulose derivative-based ChLC films, not only in situ polymerization but also casting is frequently adopted for CNC-based films. In colloidal ChLCs formed by CNCs, the length scale of the interplanar spacing of the pseudonematic planes is longer than that of molecular ChLCs formed by cellulose derivatives. As a result, the cholesteric helical pitch of the CNC ChLC dispersion remains in the visible-wavelength region, even at high concentration. The ChLC structure with concentrated dispersion is then immobilized in the obtained film after complete evaporation of the solvent, because of the low mobility of CNC rods under high concentration. Whereas cellulose derivative-based ChLC solutions can be prepared by simply dissolving dried cellulose derivatives in solvents, completely dispersing dried CNC in solvents is difficult. Therefore, CNC-based lyotropic ChLCs are prepared by concentrating dilute CNC dispersions obtained after acid hydrolysis (and purification) of cellulose. The typical concentration methods are solvent evaporation [4] and dialysis [14,45].

#### 2.1.1. Mechanochromic Films

Materials that exhibit a color change in response to mechanical stimuli—i.e., mechanochromic materials—have applications as e.g., mechanical damage sensors and energy-saving display devices. A representative approach to developing mechanochromic materials is using structural color [46]. Structurally colored materials present color that is typically ascribed to Bragg reflections from the materials’ internal periodic layered structures, and this color can change when a given mechanical stimuli varies the periodicity [47,48,49,50]. ChLCs also exhibit structural color that can change in accordance with the helical periodicity, and thus ChLCs can be key structures for constructing structurally colored mechanochromic materials. In addition, ChLCs also exhibit CD and thus optical chirality. This unique property of ChLCs may bring new concepts to the field of mechanochromic materials.

**Figure 2:**
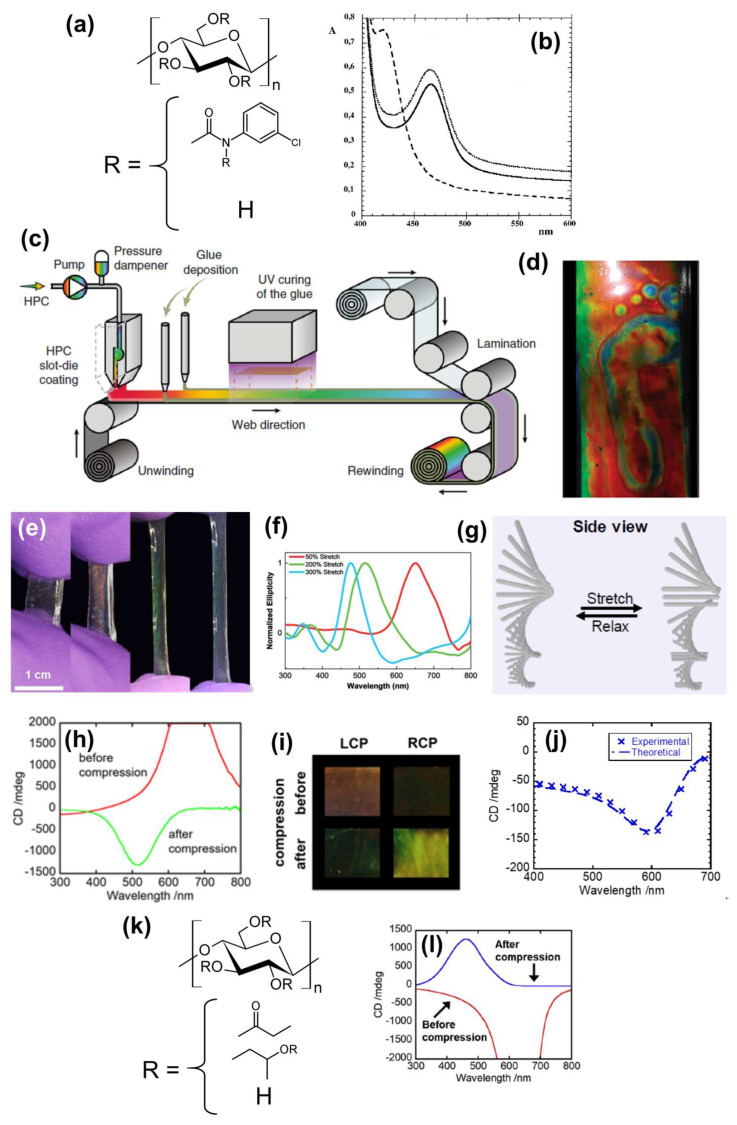
(**a**) Chemical structure of cellulose 3-chlorophenylcarbamate and (**b**) ultraviolet (UV)–visible spectra of a cholesteric liquid crystalline (ChLC) film before compression (solid line), directly after compression (dashed line), and after heating at 50 °C (dotted line) (reproduced from [51] by permission of Wiley). (**c**) Schematic of roll-to-roll preparation of hydroxypropyl cellulose (HPC) ChLC films and (**d**) footprint exhibited by the films (adapted from [52] by permission of Springer Nature). (**e**) Visual appearances and (**f**) circular dichroism (CD) spectra of a stretched cellulose nanocrystal ChLC elastomeric film. (**g**) Proposed model of a change in the ChLC structure in the film under tensile ((**e**–**g**) were reproduced from [53] by permission of Wiley). (**h**) CD spectra of ethylcellulose/poly(acrylic acid) (EC/PAA) ChLC films before and after compression at 130 °C and (**i**) visual appearance through circular polarizers (LCP: left-handed circular polarizer; RCP: right-handed circular polarizer) (reproduced from [54] by permission of the Royal Society of Chemistry). (**j**) Experimental and theoretical CD spectra of compressed EC/PAA films (adapted with permission from [56], copyright (2021) American Chemical Society). (**k**) Chemical structure of propionylated HPC (PHPC) and (**l**) CD spectra of PHPC/poly(methyl methacrylate) ChLC films before and after compression at 30 °C (reproduced from [58] by permission of Elsevier).

Müller and Zentel [51] prepared structurally colored mechanochromic films from cellulose derivative-based ChLC. They prepared lyotropic ChLC solutions by dissolving 44 wt% cellulose 3-chlorophenylcarbamate (Figure 2a) in acrylate monomer with crosslinker, and then subjecting the solutions to in situ thermal polymerization. The researchers compressed the ChLC film between two glass slides and evaluated the mechanochromic properties of the film by UV–visible (Vis) spectroscopy. The selective reflection wavelength of the ChLC film is blue-shifted by compression (Figure 2b). Furthermore, thermal treatment of the film recovered the original reflection wavelength.

Liang et al. [52] reported large-area photonic films from HPC by the roll-to-roll method. They produced the photonic films by laminating HPC in aqueous ChLC but did not conduct immobilization through solution-casting or in situ polymerization. The roll-to-roll fabrication consisted of coating the lyotropic ChLC on a polyethylene terephthalate substrate, glue-deposition to seal the edge of the film, UV-curing the glue, and laminating the film (Figure 2c). The laminated ChLC films exhibited a decrease in the reflection wavelength in accordance with increasing applied compression stress. The reflection spectra broadened by compression, probably because of misalignment of the ChLC domain induced by lateral shear flow. The researchers presented an excellent touch-sensing function of the laminated ChLC films through visualizing footprints by color change (Figure 2d).

Boott et al. [53] developed mechanochromic elastomeric films by compounding CNC-based ChLC films with synthetic polymers. They cast 4 wt% CNC aqueous suspensions to conduct EISA to obtain CNC ChLC films. The researchers then soaked the films with a dimethylsulfoxide (DMSO) solution of ethyl acrylate, 2-hydroxyethyl acrylate, and initiator to infiltrate these reagents into the films. They heated the vial to initiate polymerization of the acrylate monomers adsorbed in the films, resulting in CNC-based ChLC elastomeric films.

UV–Vis spectra of the stretched ChLC elastomeric film indicate that the film’s color blue-shifted in accordance with increasing tensile strain (Figure 2e). This shift indicates a decrease in the cholesteric helical pitch of the film by stretching. CD spectra also supported this result and exhibited a positive CD signal—demonstrating preservation of CNC-based left-handed ChLC in the stretched film (Figure 2f). Relaxation recovered the original color of the film. To evaluate the effect of the tensile stress on the internal ChLC structure, Boott et al. [53] conducted two-dimensional X-ray diffraction analyses for the relaxed and stretched sample. They observed a diffraction peak corresponding to unwinding of the ChLC structure into a pseudonematic structure when they stretched the sample. Accordingly, the researchers proposed a structural model of CNC assembly in a stretched film whereby some of the CNC rods were almost parallel to the stretch direction before stretching, and these CNC rods completely aligned with the stretched direction upon application of stress, giving rise to a small nematic region (Figure 2g).

Whereas most studies on cellulosic ChLC-based mechanochromic materials investigated force-induced color changes, Miyagi and Teramoto [54] focused on an effect of mechanical stimuli on the CD of cellulosic ChLC films. They prepared ethylcellulose/poly(acrylic acid) (EC/PAA) ChLC films by dissolving 47 wt% EC in AA solvent with photoinitiator followed by in situ photopolymerization of AA.

Miyagi and Teramoto [54] subjected 47 wt% EC/PAA ChLC films to compression at 130 °C, which is higher than the glass transition temperature (*T*_g_) of the films, and measured the CD spectra of the compressed films. The original (i.e., not compressed) EC/PAA films exhibited a positive CD signal, indicative of selective reflection of L-CPL; whereas the compressed films exhibited a negative CD signal corresponding to selective reflection of R-CPL (Figure 2h). In addition, the researchers visually inspected the EC/PAA films with circular polarizers before and after the compression. The reflected light from the original films transmitted through LCP, whereas the reflected light from the compressed films passed through RCP; which is consistent with the spectroscopic measurements (Figure 2i). These CD and circular polarization results are indicative of stress-induced circular dichroic inversion (SICDI) of the ChLC films. The researchers hypothesized that the mechanism of this circular dichroic inversion is as follows: the component polymers of the ChLC films were partially linearly oriented, resulting in birefringence of the transmitted CPL that was to undergo handedness inversion. They derived a mathematical model, taking this hypothesis into account, to calculate theoretical CD spectra of the compressed EC/PAA films by modifying the equation reported by Ritcey et al. [55]. The obtained theoretical CD spectra of the compressed films exhibited good agreement with corresponding experimental spectra (Figure 2j), indicating the validity of the researchers’ hypothesis for the mechanism of SICDI [56].

In prior work EC/PAA films expressed SICDI only above 130 °C because of the *T*_g_ of the films. Thus Miyagi and Teramoto [57,58,59] also expanded the conditions where such films can manifest SICDI. Accordingly, they prepared propionylated HPC (PHPC; Figure 2k), which has a low *T*_g_ (ca. −20 °C) and high ChLC-formability in a wide range of monomeric solvents. In this system, the ability to immobilize ChLC structures into films by in situ polymerization depended on the species of the in situ-generated polymers. Fukawa et al. [60] reported a similar phenomenon. Spin-lattice relaxation analyses of PHPC/synthetic polymer ChLC films by solid-state nuclear magnetic resonance spectroscopy indicated that the immobilization efficiency was high for the films where PHPC and synthetic polymers were not completely compatible [57,58]. This can be attributed to the way that the complete mixing of cellulose derivatives and synthetic polymers disturbs the interaction of mesogenic cellulosic chains. PHPC/synthetic polymer films with an immobilized ChLC structure exhibited SICDI by compression at room temperature (Figure 2l).

#### 2.1.2. Biomineralized Films

Living organisms build structurally strong tissues in their bodies by compounding organics and minerals—so-called organic–inorganic hybrids, such as vertebrate bones and teeth, fish scales, crustacean exoskeletons, and shells. Representative minerals are e.g., hydroxyapatite, calcium carbonate, and magnetite. Inspired by these natural structures, functional material designs based on biomineralization are an active area of research [61,62]. There has been research on biomineralization of cellulosic ChLC films, as discussed next.

Ogiwara et al. [63] conducted calcium phosphate biomineralization using a cellulose derivative-based ChLC as an organic scaffold. This concept likely mimics the mineralized supramolecular helical structure that is seen in the crustacean exoskeleton. Although they investigated EC and HPC-based ChLC films, we focus on only the EC system. The researchers prepared EC/PAA films by in situ photopolymerization of 40–57 wt% EC/AA ChLC solutions and soaked the obtained films in mineral solutions containing CaCl_2_, (NH_4_)_2_HPO_4_, and NaCl at pH 9.0. They then rinsed the mineralized films with water and vacuum-dried the films. In this material design, PAA is a crucial component that has the two roles of immobilizing the cellulosic ChLC structure and absorbing the ions, thus facilitating mineralization on the ChLC scaffold.

**Figure 3:**
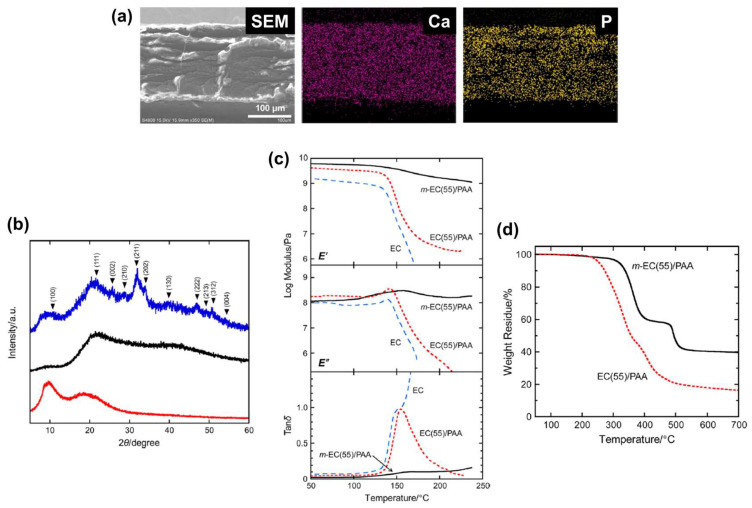
(**a**) Scanning electron microscope (SEM) image and energy-dispersive X-ray mappings of Ca and P for the fracture surface of an *m*-EC(55)/PAA film. (**b**) Wide-angle X-ray diffraction intensity profiles of EC(55)/PAA (bottom), *m*-EC(55)/PAA (center), and p-*m*-EC(55)/PAA film (top). (**c**) Temperature dependence of the storage modulus *E*’, loss modulus *E*’’, and loss factor tan *δ* for EC (dashed line), EC(55)/PAA (dotted line), and *m*-EC(55)/PAA film (solid line). (**d**) Thermogravimetric analysis profiles for EC(55)/PAA and *m*-EC(55)/PAA films ((**a**–**d**) were reprinted with permission from [63], Copyright (2021) American Chemical Society).

Energy-dispersive X-ray analyses of the fracture surface of mineralized 55 wt% EC/PAA films (*m*-EC(55)/PAA) indicate that the elements Ca and P were uniformly distributed inside the films by mineralization (Figure 3a). Wide-angle X-ray diffraction profiles of unmodified 55 wt% EC/PAA films (EC(55)/PAA; Figure 3b) showed a diffraction peak that is attributable to the stacked pseudonematic planes of ChLC at *θ* = 9.6° and a halo corresponding to an amorphous region at *θ* ≈ 19°. Both the unmodified and as-mineralized EC/PAA films exhibited no clear crystalline diffraction pattern, whereas the mineralized films subjected to posttreatment in aqueous NaOH (p-*m*-EC(55)/PAA) exhibited diffraction peaks from crystalline hydroxyapatite (Figure 3b). The NaOH posttreatment results are probably attributable to alkali hydrolysis of amorphous calcium phosphate or less-ordered precursors of hydroxyapatite.

In dynamic mechanical analyses of the film samples, the storage and loss modulus of EC(55)/PAA films substantially diminished from 140 °C, whereas those of *m*-EC(55)/PAA films retained relatively high values even above 200 °C (Figure 3c). Thermogravimetric analyses of these samples indicate that the temperature at which polymer degradation began and the residual weight at 700 °C for *m*-EC(55)/PAA films were higher than those of EC(55)/PAA films (Figure 3d). These dynamic mechanical analysis and thermogravimetric analysis results reveal that biomineralization enhanced the thermomechanical properties and thermal stability of EC/PAA ChLC films.

Katsumura et al. [64] and Nakao et al. [65] reported calcium carbonate mineralization of EC/PAA, CNC/poly(2-hydroxyethyl methacrylate), and CNC/poly(HEMA-*co*-AA) ChLC films. All of these systems exhibited improved thermomechanical properties and thermal stability, as well as calcium phosphonate mineralization system.

#### 2.1.3. Optical Filters

Although ChLC can act as an optical filter to separate the CPL of particular wavelengths and handedness, this reflection-selectivity is sometimes too specific. However, by combining ChLC with a nematic liquid crystalline phase or substances that affect the cholesteric helical structure, the reflection-selectivity can be expanded. This enables fabrication of optical filters with a filtering function tailored to assumed applications.

**Figure 4:**
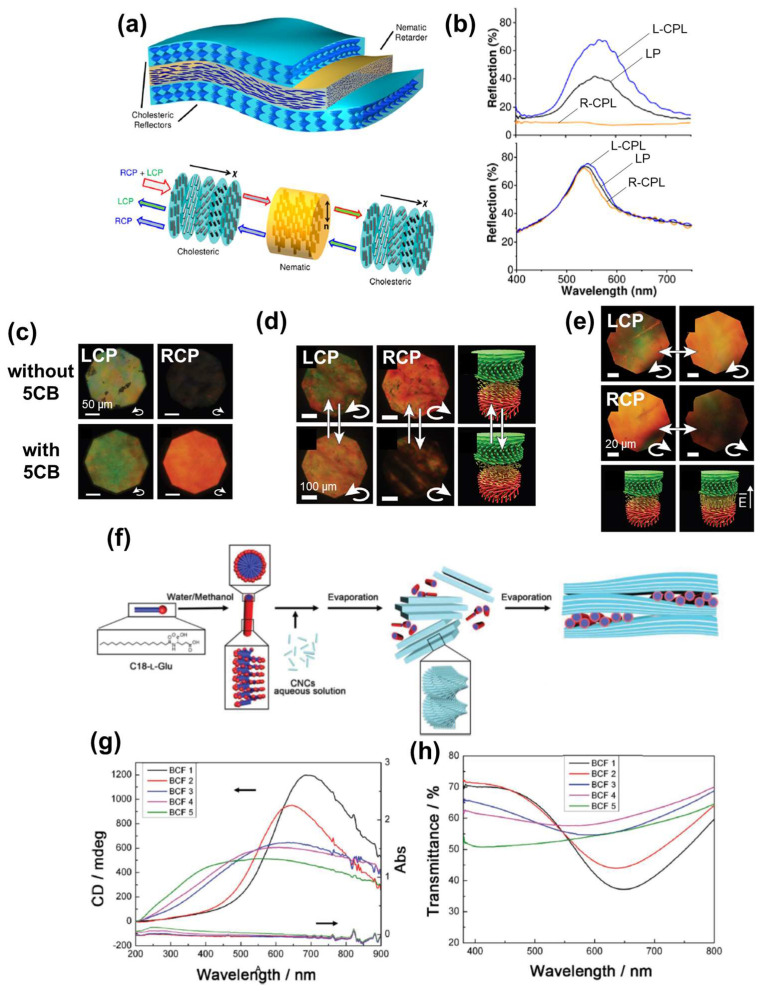
(**a**) Schematic describing the sandwich film composed of cellulose nanocrystal (CNC)-based cholesteric liquid crystal (ChLC) outer layers and a nematic inner layer, and the mechanism of the handedness-independent circularly polarized light (CPL) reflection. LCP, left-handed circular polarizer; RCP, right-handed circular polarizer. (**b**) Reflection spectra of a single CNC ChLC film (top) and a sandwich film (bottom) with linearly polarized (LP) light, right-handed CPL (R-CPL), and left-handed CPL (L-CPL) incident light ((**a**,**b**) were reprinted with permission from [66], copyright (2021) American Chemical Society). (**c**) Reflection images of a simple CNC ChLC film and a CNC–4-pentyl-4′-cyanobiphenyl (5CB) composite through LCP and RCP. (**d**) Reflection image of a CNC–5CB composite through LCP and RCP at 30 °C (top) and 34.5 °C (bottom). (**e**) Reflection image of a CNC–5CB composite through LCP and RCP; before (left) and after (right) applying an electric field parallel to the cholesteric helical axis ((**c**–**e**) were reproduced from [67] by permission of Wiley). (**f**) Schematic presenting formation of the ChLC structure with a distributed helical pitch in the presence of *N*-stearoyl-L-glutamic acid (C18–L-Glu micelles). (**g**) Circular dichroism spectra and (**h**) transmission spectra of broadband cellulose films (BCFs) with various portions of C18–L-Glu ((**f**,**g**) were reproduced from [68] by permission of Wiley).

De la Cruz et al. [66] produced optical filters that reflect both L-CPL and R-CPL with a certain wavelength, by combining ChLC films and nematic liquid crystalline films, both prepared from CNCs. These filters have a reflection-selectivity for wavelength and no selectivity for handedness. The researchers designed the optical filters by a sandwich structure with top and bottom left-handed ChLC CNC layers, and a middle nematic CNC layer as a wave-plate (Figure 4a). In this system, the first ChLC layer selectively reflected L-CPL, and the birefringence inverted the transmitted R-CPL in the middle nematic layer. The L-CPL thus generated was reflected from the second ChLC layer, inverted back into R-CPL in the middle layer, and transmitted through the first ChLC layer. This mechanism is analogous to the SICDI by the compressed EC/PAA ChLC films described in Section 2.1.1.

De la Cruz et al. [66] prepared CNC-based ChLC films by curing mixtures of a CNC aqueous dispersion, 1,2-bis(trimethoxysilyl)ethane (organosilica precursor), and low-molecular-weight polyethylene glycol (plasticizer) in ambient conditions. Curing was in the presence of a static magnetic field normal to the film surface for uniform orientation of the helical axis: i.e., perpendicular to the film surface. The helical pitch, and thus a selective reflection wavelength, of the films can be tuned by varying the feed ratio of the CNC aqueous dispersion, organosilica, and polyethylene glycol. The researchers prepared CNC-based nematic films by linear shear deposition of a 3.5–5.0 wt% CNC aqueous dispersion on the ChLC films, and subsequent drying at 30 °C to 35 °C. They prepared sandwich films by attaching the ChLC film with the nematic layer to the pristine ChLC film with an adhesive.

UV–Vis reflection spectroscopy of the single ChLC CNC layer exhibited no reflection of R-CPL because of the selective reflection of L-CPL. In addition, the reflectance of linearly polarized (LP) light was half that of L-CPL because LP light is the vector sum of R-CPL and L-CPL (Figure 4b). In contrast, the sandwich film exhibited almost the same reflectivity for LP light, L-CPL, and R-CPL; indicating the handedness-independent reflection of CPL (Figure 4b). This interpretation is supported by CD spectra that exhibited no peak for the sandwich film because of equal reflection of both L-CPL and R-CPL, and transmission spectra where the minimum transmittance of the sandwich film was half that of the single-ChLC film. Such handedness-independent optical filters can achieve full reflection of both L-CPL and R-CPL, enabling twice the efficiency of the resulting photoenergy in comparison with single-ChLC films.

Fernandes et al. [67] fabricated CNC ChLC films that also reflect both R-CPL and L-CPL, by incorporating a small-molecule nematic liquid-crystal in the films. Although the concept of this material to accomplish handedness-independent reflection is the same as in de la Cruz et al. [66], their material designs are different. Whereas de la Cruz et al. [66] constructed the optical filters from completely separated ChLC films and nematic liquid crystalline films, Fernandes et al. [67] integrated the ChLC films and nematic liquid-crystal as one material system.

Fernandes et al. [67] prepared ChLC films by casting a 1.9 wt% CNC aqueous dispersion on a Petri dish and drying the dispersion at 20 °C for 4 w to allow EISA. They prepared sample cells by placing the ChLC films between two glass slides and filled the sample cells with the nematic liquid-crystal 4-pentyl-4′-cyanobiphenyl (5CB) to obtain CNC–5CB composite liquid crystalline films.

Fernandes et al. [67] observed the pristine CNC ChLC films and the CNC–5CB composite films with circular polarizers. Whereas the CNC films exhibited the reflection color in the LCP image but no color in the RCP image, the composite films exhibited the reflection color in both the LCP and RCP images (Figure 4c). These differing reflection results indicate that the composite films reflected both L-CPL and R-CPL. Analogously to de la Cruz et al.’s [66] strategy, the birefringence in the nematic region inverted the R-CPL transmitted through the outer ChLC region, reflected this inverted transmission from the inner ChLC region, reinverted this reflection as R-CPL in the nematic region, and transmitted the reinversion through the outer ChLC region.

Because 5CB is a small-molecule liquid crystal, it has high stimuli-sensitivity and a fast response. Taking advantage of this characteristic, the researchers can modulate the reflection properties of CNC–5CB composites by external stimuli—such as the temperature and electric field. The composite films exhibited handedness-independent reflection of CPL at 30 °C, whereas they exhibited selective reflection of L-CPL at 34.5 °C (Figure 4d). This is attributable to 5CB not exhibiting birefringence because of the nematic–isotropic phase transition at ~35 °C. Applying an electric field parallel to the cholesteric helical axis eliminated the reflection of R-CPL (Figure 4e), because no birefringence occurs when the orientation direction of an anisotropic material is parallel to the light direction. The CNC–5CB composite is promising for applications as an optical filter that can dynamically switch their filtering mode.

Cao et al. [68] developed optical filters that reflect L-CPL with a wide range of wavelengths in the visible region, by combining ChLC films with a surfactant. These films have reflection-selectivity for the handedness and no selectivity for the wavelength. The concept of the material design is that nanometer-scale micelles formed from the surfactant disturb the self-assembly of CNC to give a nonuniform cholesteric helical pitch, resulting in broadband reflection of L-CPL (Figure 4f).

Cao et al. [68] obtained CNC-based ChLC films compounded with micelles by adding a controlled quantity of micelle solution composed of a water/methanol mixture and anionic surfactant *N*-stearoyl-L-glutamic acid (C18–L-Glu) into a 4 wt% CNC aqueous dispersion at neutral pH, followed by EISA at room temperature for 3 d. The researchers termed the prepared films as broadband cellulose films (BCFs).

Figure 4g shows CD spectra of BCFs with various proportions of C18–L-Glu (BCF1–BCF5; the C18–L-Glu content increases from BCF1–BCF5). These spectra indicate that the reflection peak of the films broadened in accordance with increasing C18–L-Glu concentration. The sample with the highest content of C18–L-Glu in the researchers’ experiments (BCF5) exhibited a broad reflection peak covering the visible region. CPL transmission spectra verified the prevention of the transmission of L-CPL over a wide range of wavelengths by ChLC films incorporating C18–L-Glu (Figure 4h). Such ChLC films that exhibit broadband selective reflection of CPL can be applied to optical filters that separate R-CPL and L-CPL regardless of wavelength.

#### 2.1.4. Conducting Films

**Figure 5:**
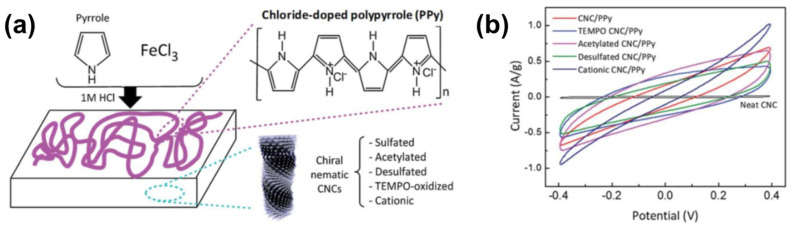
(**a**) Schematic for preparing cellulose nanocrystal (CNC)/polypyrrole (PPy) composite films. (**b**) Cyclic voltammograms of CNC/PPy composites, where the CNCs were subjected to various surface modifications ((**a**,**b**) were reproduced from [69] by permission of Wiley). TEMPO, (2,2,6,6-tetramethylpiperidin-1-yl)oxyl.

Lizundia et al. [69] fabricated conducting materials by depositing a conducting polymer, polypyrrole (PPy), on CNC ChLC films. They prepared the ChLC films by casting 4 wt% CNC aqueous dispersions on Petri dishes, and then air-drying the dispersions for 96 h to induce EISA. To prepare CNC ChLC films with various functional groups on the surfaces, the researchers conducted surface modification of the films by (2,2,6,6-tetramethylpiperidin-1-yl)oxyl–oxidation, acetylation, alkaline desulfation, and cationization. They obtained CNC/PPy composite films by in situ oxidative polymerization of pyrrole on a series of CNC films as substrates (Figure 5a). The researchers soaked the CNC films in distilled water; then added pyrrole, aqueous FeCl_3_ as an oxidant, and HCl as a dopant, before finally conducting the reaction at 0 °C for 5 h.

Lizundia et al. [69] performed cyclic voltammetry using CNC/PPy composite films as electrodes to characterize the conductivity of the films (Figure 5b). Whereas the neat CNC film without PPy exhibited zero current over the range of applied voltage, CNC/PPy composite films exhibited rectangular cyclic voltammetry curves. The specific capacitance calculated from the voltammogram of the acetylated CNC/PPy film was 4× that of the unmodified CNC/PPy film. These cyclic voltammetry results indicate that the capacitance can be improved by surface modification of composite films. The researchers considered this improvement to be related to the hydrogen bonding between the carbonyl and hydroxy groups on the CNC film, and the amine groups of the PPy.

### 2.2. Cellulosic ChLC Gels

A polymeric gel is composed of a polymer network constructed by chemical or physical crosslinking of the polymer chains, and swells by uptaking solvents into the network. Although Section 2.1 introduced some film materials—such as the mechanochromic films created by Müller and Zentel [51] and the biomineralized films reported by Ogiwara et al. [63] that had a polymer network structure, these materials express their functions in a dried state. We consider materials (incorporating cellulosic ChLC) that express their functions through swelling with a solvent as cellulosic ChLC functional gels.

Because cellulosic ChLC can be formed in an aqueous solution or a dispersion using HPC or CNC, these cellulosics enable production of ChLC hydrogels. This hydrogel characteristic facilitates fabrication of ecofriendly, biocompatible, cellulosic ChLC-based functional gels.

Researchers prepare cellulose derivative-based ChLC gels by in situ polymerization of lyotropic ChLC solutions of cellulose derivatives in monomeric solvents with a crosslinker [70]. Another method is formation of lyotropic ChLC solutions of cellulose derivatives with crosslinkable side groups followed by crosslinking [51]. A representative preparation of CNC-based ChLC gels is as follows: first mix a relatively dilute non-ChLC dispersion with a crosslinkable monomer, and then subject the mixture to EISA and in situ polymerization [71,72].

#### 2.2.1. Salt-Responsive Color Gels

Chiba et al. [70] reported cellulose derivative-based ChLC hydrogels that exhibit a salt-responsive color change. They prepared lyotropic ChLC by dissolving 60–72 wt% HPC in a solvent mixture composed of di(ethylene glycol) monomethyl ether methacrylate (DEGMEM), methanol, water, and tetra(ethylene glycol) diacrylate. The researchers added glutaraldehyde and a small quantity of hydrochloric acid into the lyotropic ChLC, followed by in situ photopolymerization and crosslinking to obtain the ChLC gels. In this gel system, they immobilized the ChLC structure with an interpenetrating polymer network consisting of HPC crosslinked with glutaraldehyde, and poly(DEGMEM) (PDEGMEM) crosslinked with tetra(ethylene glycol) diacrylate.

**Figure 6:**
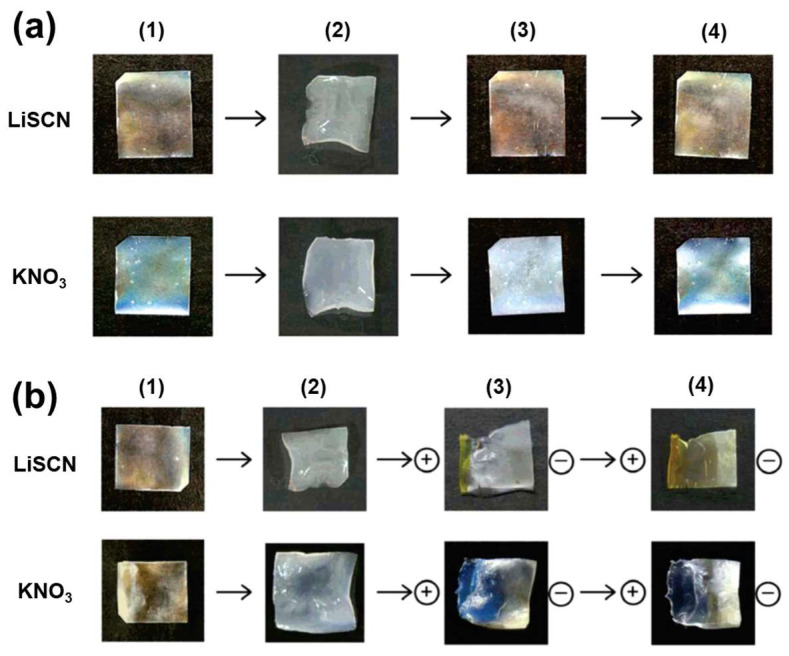
(**a**) Visual appearance of hydroxypropyl cellulose (HPC)/poly(di(ethylene glycol) monomethyl ether methacrylate) (PDEGMEM) gels (1) in the original state, (2) swollen in salt solutions, (3) dried after salting-in, and (4) washed with water followed by drying. The researchers used LiSCN and KNO_3_ as salts. (**b**) Visual appearance of HPC/PDEGMEM gels (1) in original state, (2) swollen in salt solutions, (3) after applying the electric field, and (4) dried after turning off the electric field ((**a**,**b**) are reprinted with permission from [70], copyright (2021) American Chemical Society).

The 65 wt% HPC/PDEGMEM composites were orange in the original dried state, whereas the color redshifted by swelling in aqueous LiSCN and subsequent drying (Figure 6a). Chiba et al. [70] restored the original color by washing the gels with distilled water and drying. However, after immersing these gels in aqueous KNO_3_ the reflected color of the gels blueshifted. The researchers explained this salt-dependent color variation in terms of the chaotropic effect of the salt ions. Because chaotropic ions act as water-structure breakers (and antichaotropic ions act as water-structure formers), the salts likely affected the water-solubility of HPC, inducing an increase in the cholesteric helical pitch in the context of chaotropic ions and a decrease in the cholesteric helical pitch in the context of antichaotropic ions. Ions with a stronger chaotropicity corresponded to a larger change in the helical pitch.

In addition, Chiba et al. [70] achieved color control of the gels with an electric field (Figure 6b). After applying an electric field to HPC/PDEGMEM gels salted with LiSCN, negative thiocyanate ions migrated to the positive side of the gels, and the coloration of the positive side redshifted because of the chaotropicity of thiocyanate.

#### 2.2.2. Humidity-Responsive Color Gels

**Figure 7:**
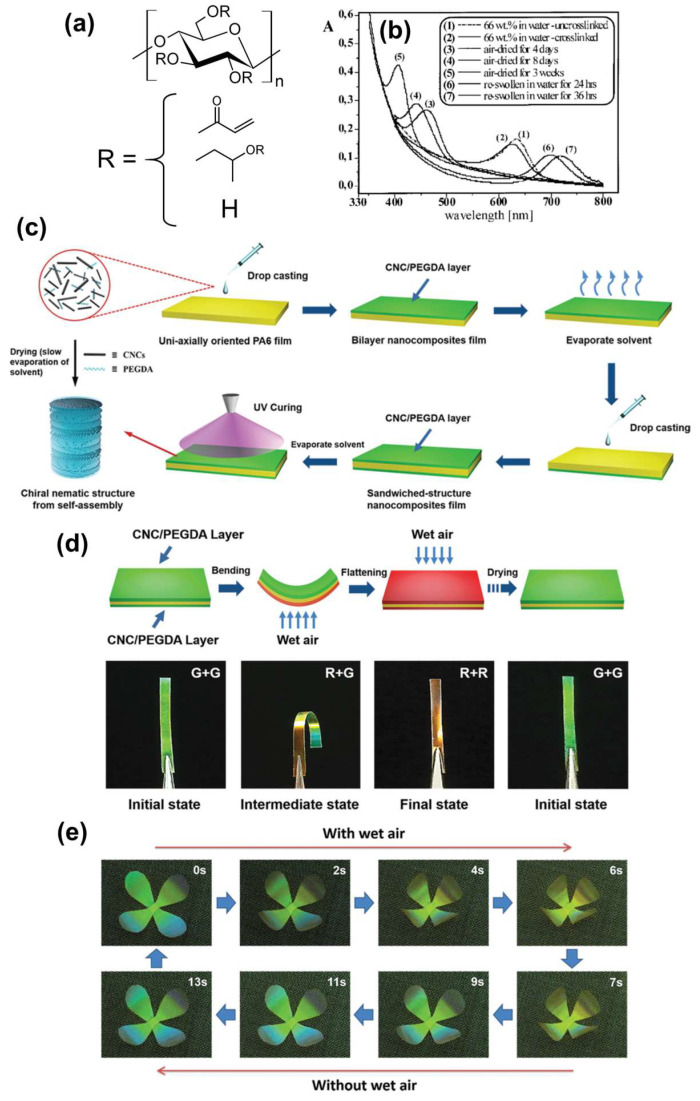
(**a**) Chemical structure of hydroxypropyl cellulose (HPC) acrylate and (**b**) effect of swelling and drying on UV–Vis spectra of an HPC-based cholesteric liquid crystal (ChLC) hydrogel (Reprinted from [51] by permission of Wiley). (**c**) Schematic of cellulose nanocrystal (CNC) ChLC gel/polyamide-6 (PA-6) film sandwich composites. PEGDA, polyethylene glycol diacrylate. (**d**) Schematic and visual appearances of water-vapor-induced color change as well as actuation of the sandwich composite. R: red; G: green. (**e**) Wet-air response of an artificial flower prepared from a sandwich composite (**c**–**e**) were reproduced from [71] by permission of The Royal Society of Chemistry).

Müller and Zentel [51] produced cellulosic molecular ChLC hydrogels that changed color in accordance with the water content. They introduced crosslinkable acryl groups into HPC by an esterification reaction between acryloyl chloride and the hydroxy groups of HPC (Figure 7a). The researchers set a relatively low degree of substitution (0.22) to maintain the water solubility. They dissolved the modified HPC in water at 66 wt% to obtain lyotropic aqueous ChLC, and then subjected the solutions to photocrosslinking to prepare ChLC hydrogels.

The obtained HPC-based ChLC hydrogel originally exhibited a red color, whereas the color blueshifted after air-drying for 3 d and shifted to the UV region after 3 w (Figure 7b). These color shifts are likely because shrinking the hydrogel by air-drying shrank the cholesteric helix incorporated in the hydrogel. The coloration of the gel returned to the red region after swelling with water, and Müller and Zentel [51] cycled the color change between the red to UV regions.

Wu et al. [71] fabricated composites (of a CNC-based ChLC gel and a polyamide film) that exhibit a humidity-responsive color change and three-dimensional deformation. They produced the composite by sandwiching a uniaxially oriented polyamide film between two CNC ChLC gel layers.

Wu et al. [71] mixed a 1.5 wt% CNC aqueous dispersion with 5 wt% aqueous polyethylene glycol diacrylate (PEGDA) to reach several weight ratios of CNC and PEGDA. They used PEGDA to construct a polymer-network structure through polymerization and to impart flexibility to the resultant polymerized product. The researchers added the CNC/PEGDA mixture to one side of a uniaxially oriented polyamide-6 (PA-6) film, and then dried the product. They subjected another side of the PA-6 film to the same process and exposed the sample to UV-irradiation for photopolymerization, resulting in a CNC ChLC gel/PA-6 film composite (Figure 7c).

Wu et al. [71] evaluated the humidity response of a specimen cut from the composite with a CNC/PEGDA mass ratio of 54.5/45.5 along the orientational axis of the PA-6 film. They placed the sample in a 10-mL bottle with water, and provided wet air to the sample by heating the bottle to 75 °C. The humidity-response tests indicate that the color of the side of the sample exposed to wet air redshifted, whereas the other side exhibited no color change (Figure 7d). In addition, the sample exhibited a bending deformation in response to the humid environment. These color-change and deformation phenomena are attributable to asymmetric swelling of the CNC-based ChLC gel outer layers, originating from a blockage of the water vapor by the hydrophobic PA-6 interlayer film. The researchers flattened the composite by wetting both sides and observed the original color after drying. They also prepared an artifact mimicking a four-petal flower from the sandwich composite and demonstrated the composite’s humidity-responsive behavior (Figure 7e). Providing wet air to the flower mimic gave rise to folding due to the bending force of the petals, whereas removing the moisture source caused recovery of the original shape by water desorption. A composite material expressing humidity-responsive color change as well as three-dimensional deformation is attractive as a potential application in, for example, humidity sensors or wet air-driven actuators.

The humidity response of CNC-based ChLC gels is an active area of research [73,74,75,76,77]. The substantial hydrophilicity and useful coloration properties of CNC-based ChLCs are suitable for achieving a humidity response.

#### 2.2.3. Colored Gels with Tailored Stimuli-Responsivity

Kelly et al. [72] produced stimuli-responsive photonic hydrogels incorporating a CNC ChLC structure. They concentrated 3 wt% CNC aqueous dispersions containing the monomers, crosslinker, and photoinitiator 2,2-diethoxyacetophenone to >60 wt% CNC, to obtain ChLC dispersions with visible color through EISA. The researchers then prepared photonic hydrogels by in situ photopolymerization of the monomer in the colored ChLC dispersions for 1 h. They used several types of monomer and crosslinker for preparing the hydrogels, such as acrylamide, *N*-isopropylacrylamide, polyethylene glycol methacrylate, *N*,*N*’-methylenebisacrylamide, and polyethylene glycol dimethacrylate.

**Figure 8:**
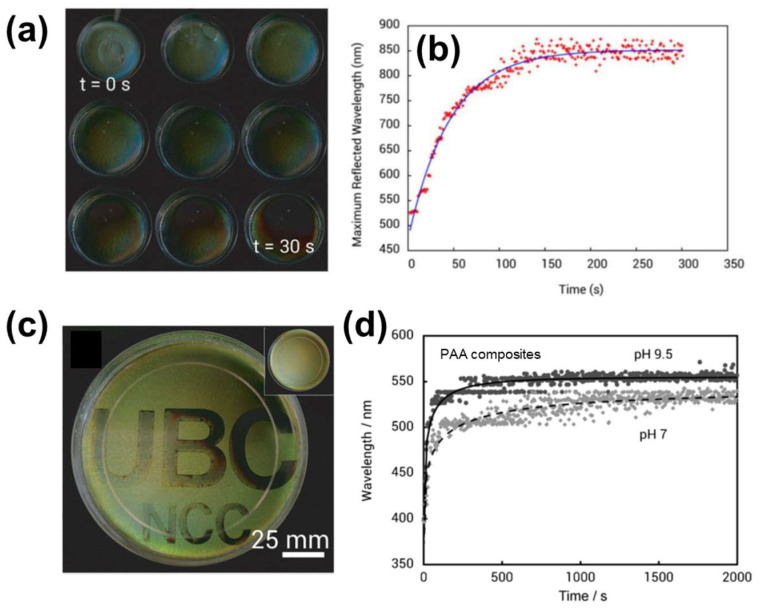
(**a**) Visual appearance of a cellulose nanocrystal (CNC)/polyacrylamide (PAAm) cholesteric liquid crystal (ChLC) hydrogel swollen in water for 30 s. (**b**) Time dependence of the selective reflection wavelength of a CNC/PAAm ChLC hydrogel swollen in water. (**c**) Swollen state in water for a CNC/PAAm ChLC hydrogel prepared by in situ photopolymerization of the precursor ChLC dispersion with a photomask. (**d**) Time dependence of the selective reflection wavelength of a CNC/poly(acrylic acid) (PAA) ChLC hydrogel swollen in water at pH 7 and 9.5 (**a**–**d**) were reproduced from [72] by permission of Wiley).

The color of the CNC/polyacrylamide hydrogels was red-shifted after swelling in water because the CNC was diluted inside the gels (Figure 8a). Immersing the hydrogels in water corresponded to rapid swelling, reaching the swelling equilibrium after ca. 150 s (Figure 8b). Hydrogels briefly subjected to photopolymerization exhibited faster swelling than those that were sufficiently irradiated because of the loose polymer network in the former. Taking advantage of this property (controllable swelling rate), Kelly et al. [72] spatially modulated the swelling behavior by masking a section of the hydrogels during photopolymerization. Such photonic hydrogels can be used to produce a security device that exhibits a latent image by swelling in water (Figure 8c).

Because diverse monomers can be used to prepare CNC-based ChLC hydrogels, a wide range of functionality can be imparted to the hydrogels by simply changing the monomer species in the aqueous dispersions. To demonstrate this functionalization strategy, Kelly et al. [72] prepared hydrogels composed of CNC and poly(acrylic acid) (PAA), and demonstrated a pH-responsive color change originating from pH-dependent ionization of PAA (Figure 8d).

### 2.3. Cellulosic ChLC Mesoporous Materials

Constructing a mesoporous structure can impart valuable physical properties to cellulosic ChLC materials, such as flexibility and substantial solvent absorption. Furthermore, chiral mesoporous structures will facilitate fabrication of unprecedented functional materials, such as chiral reaction fields and optical resolution columns.

#### 2.3.1. Flexible and Solvent-Responsive Mesoporous Films

**Figure 9:**
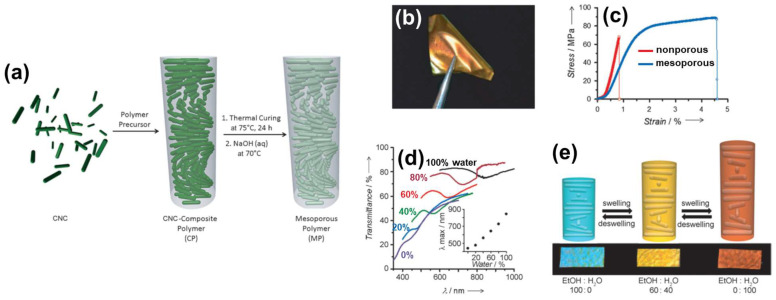
(**a**) Schematic for preparing mesoporous cholesteric liquid crystal (ChLC) plastics from cellulose nanocrystal (CNC)/phenol–formaldehyde (PF) resin ChLC dispersions. (**b**) Visual appearance of a mesoporous ChLC plastic deformed by bending. (**c**) Stress–strain curves of a nonporous CNC/PF resin composite film and a mesoporous ChLC plastic. (**d**) Ultraviolet–visible transmission spectra of mesoporous ChLC plastics immersed in a water/ethanol mixture. (**e**) Schematic of the swelling behavior of mesoporous ChLC plastics in water/ethanol mixtures (**a**–**e**) were reproduced from [78] by permission of Wiley).

Khan et al. [78] produced flexible mesoporous ChLC films by compounding CNC with phenol–formaldehyde (PF) resin. In a typical procedure, they mixed a 3.5 wt% CNC aqueous dispersion with 35 wt% PF solution in water/methanol, and then dried the mixture under ambient conditions for EISA. The researchers heated the obtained films at 75 °C for 24 h to cure the PF resin, resulting in CNC/PF resin composite films. They prepared mesoporous films upon treatment of the composites with an alkali solution or an alkali/urea mixture to remove the internal CNC, followed by supercritical drying with CO_2_ (Figure 9a).

Khan et al. [78] retained coloration of the films, derived from selective reflection of CPL, even after removal of the CNCs. This retained coloration indicates that the researchers obtained a CNC-based ChLC morphology on the mesoporous structure. In addition, the mesoporous ChLC films were pliable and readily bent without structural damage (Figure 9b), in contrast to the brittle nature of composites before removing the CNCs. Tensile stress–strain curves indicate that the mesoporous films exhibited higher flexibility (lower elastic modulus) and toughness (higher elongation at break) than the corresponding nonporous films (Figure 9c). The researchers also examined the solvent-responsivity of the mesoporous ChLC films. The reflection color of the film red-shifted upon immersing the film in a water/ethanol mixed solvent, and the color corresponded to the water/ethanol ratio (Figure 9d). This is attributable to an increase in the cholesteric helical pitch originating from swelling of the mesoporous structure (Figure 9e). The color change was reversible by deswelling. Such CNC-based mesoporous ChLC films may find use as e.g., structurally colored plastics and solvent sensors.

#### 2.3.2. Cellulose Derivative–Silica Mesoporous Hybrids for Chiral Chromatography

**Figure 10:**
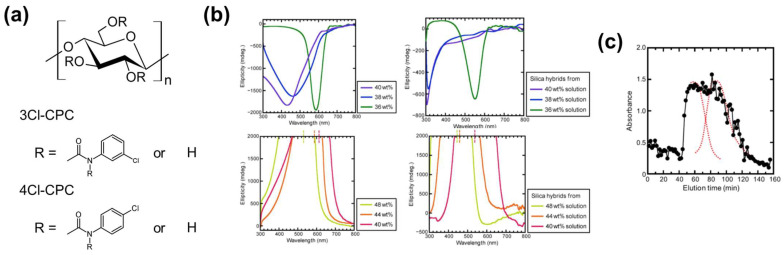
(**a**) Chemical structures of cellulose, 3-chlorophenylcarbamate (3Cl-CPC), and 4-chlorophenylcarbamate (4Cl-CPC). (**b**) Circular dichroism spectra of 3Cl-CPC/3-aminopropyltrimethoxysilane (left) and 4Cl-CPC/tetramethyl orthosilicate/dimethylformamide/dichloroacetic acid (right) cholesteric liquid crystal solutions with various cellulosic concentrations. (**c**) Chromatographic profile for racemic *trans*-stilbene oxide using an open column filled with a 4Cl-CPC–silica hybrid (**a**–**c**) were reproduced from [79] by permission of Elsevier).

Sato et al. [79] prepared cellulose chlorophenylcarbamate derivative–silica hybrids with ChLC as well as mesoporous structures by a sol–gel process, and evaluated their optical and chiral resolution properties. They added 3- and 4-chlorophenyl isocyanate into 2.8 wt% cellulose in dimethylacetamide/LiCl, and carried out the reaction at various temperatures and times under nitrogen. The researchers purified the obtained cellulose 3-chlorophenylcarbamate (3Cl-CPC) and 4-chlorophenylcarbamate (4Cl-CPC; Figure 10a) by dissolution–precipitation and Soxhlet extraction. They prepared lyotropic ChLC solutions of 3Cl-CPC by dissolving 3Cl-CPC in 3-aminopropyltrimethoxysilane at 32–40 wt%, and prepared ChLC solutions of 4Cl-CPC by dissolving 4Cl-CPC in a tetramethyl orthosilicate/dimethylformamide/dichloroacetic acid mixed solvent at 32–48 wt%. The researchers exposed the ChLC solutions of 3Cl-CPC and 4Cl-CPC to air to solidify the solutions through the sol–gel process, resulting in cellulose chlorophenylcarbamate–silica hybrids.

In CD spectroscopy, the concentrated 3Cl-CPC/3-aminopropyltrimethoxysilane solutions exhibited negative peaks originating from the right-handed cholesteric helix, whereas 4Cl-CPC/tetramethyl orthosilicate/dimethylformamide/dichloroacetic acid solutions exhibited positive peaks originating from a left-handed helix (Figure 10b). Sato et al. [79] also observed the selective reflection peaks in CD spectra of silica hybrids obtained by sol–gel processing of those ChLC solutions, indicating immobilization of the ChLC structure in the hybrids (Figure 10b). The reflection peaks of the solidified samples blue-shifted compared with those of the corresponding parent solutions, likely because the samples shrank during gelation.

Sato et al. [79] attempted a chiral resolution of a racemic compound by column chromatography with the cellulose chlorophenylcarbamate–silica ChLC hybrid as the column filler. A concept of this column system is two-chirality with different scales, which are the molecular chirality of the cellulose derivative and the supramolecular chirality of the ChLC structure; whereas conventional chiral columns adopt only molecular chirality. The researchers carried out an open-column chromatographic separation of a racemate of *trans*-stilbene oxide (TSO) into two enantiomers [i.e., (*R*,*R*)-TSO and (*S*,*S*)-TSO] with a column filled with 4Cl-CPC–silica ChLC hybrid granules (*φ* ≤~120 μm). The resultant chromatogram exhibited a broad peak composed of two signals (Figure 10c), although a conventional chiral column manufactured from 4Cl-CPC enable chiral separation of TSO. The researchers attributed this insufficient chiral resolution by 4Cl-CPC–silica ChLC hybrid to fewer contacts of the filler with the chiral molecules, itself attributable to the nonuniform particle size of the filler. Technical optimization will validate the concept of two-chirality with different scales.

### 2.4. Other Cellulosic ChLC Solid Functional Materials

There are additional reports on diverse cellulosic ChLC solid functional materials—such as structurally colored liquid marbles [80] and clay composites [81], photonic skins to visualize human motion [82,83], transistors for CPL-sensing [84], solvent-resistant photonic films [85], mechanically anisotropic composites [86], CPL-luminescent films incorporating quantum dots [87], pressure-sensitive aerogels [88], humidity-responsive photonic microarrays [89], and inkjet-printed photonic patterns [90]. These studies will facilitate expansion of the range of applications of cellulosic materials to industrial sectors in which cellulose is often considered to be incompatible with high performance.

## 3. Cellulosic ChLC-Based Liquid Functional Materials

### 3.1. Cellulosic ChLC Solutions

A representative material form of cellulosic ChLC liquid materials is solution; that is, lyotropic ChLC. Although the basic physical properties of cellulosic ChLC solutions have been thoroughly investigated to date, research on functionalization of the solutions have been limited. A core reason for this lack of research may be the high viscosity of cellulosic ChLC solutions, which leads to handling difficulties and slow stimuli-responses.

Nishio et al. [91] and Chiba et al. [92] reported cellulose derivative-based ChLC solutions doped with salts and modulated the solutions’ color with an electric field. This system is a precursor of the salt-responsive color gels described in Section 2.2.1.

**Figure 11:**
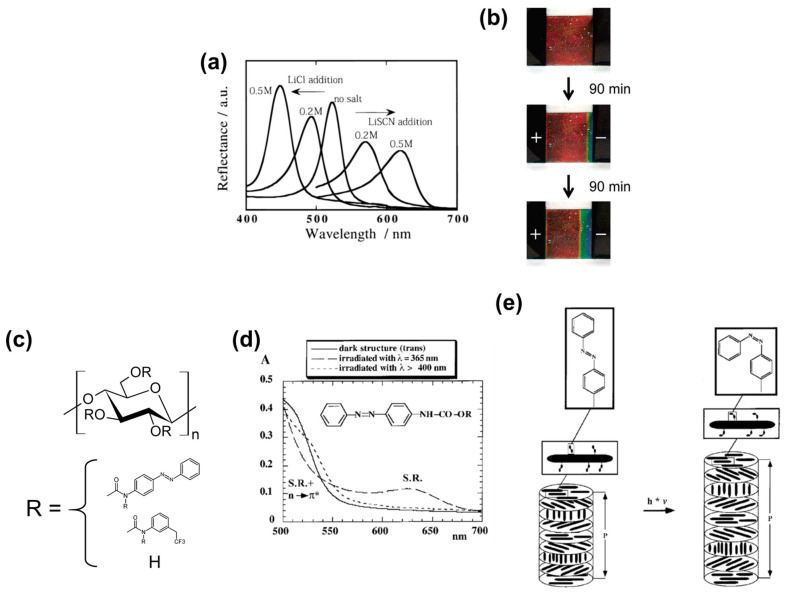
(**a**) Ultraviolet–visible (UV-Vis) spectra of hydroxypropyl cellulose (HPC) cholesteric liquid crystal (ChLC) aqueous solutions with various concentrations of LiCl and LiSCN. (**b**) Visual appearance of HPC ChLC aqueous solutions with LiSCN under application of an electric field (**a**,**b**) were reprinted with permission from [91,92]. Copyright (2021) American Chemical Society). (**c**) Chemical structure of azo-cellulose. (**d**) UV–Vis spectra of an azo-cellulose ChLC solution prior to irradiation, after irradiation at 356 nm, and after irradiation at >400 nm. (**e**) Schematic of the change in the cholesteric helical pitch of azo-cellulose ChLC solutions by irradiation (**d**,**e**) were reproduced from [51] by permission of Wiley).

Nishio et al. [91] prepared ChLC solutions by dissolving HPC into aqueous salt. They accelerated dissolution of HPC by repeated centrifugation of the mixture. The selective reflection wavelength of 62.5 wt% HPC ChLC aqueous solution blue-shifted in accordance with increasing concentration of LiCl, whereas the wavelength red-shifted in accordance with increasing concentration of LiSCN (Figure 11a). This difference is likely related to the chaotropicity of the salt ions as aforementioned in Section 2.2.1. Chiba et al. [92] applied an electric field to HPC ChLC aqueous solutions containing LiSCN, and found that the positive electrode side of the solutions red-shifted and the negative electrode side blue-shifted (Figure 11b). Which electrode side exhibited a blue-shift and which side exhibited a red-shift depended on the salt species. A long time (~3 h) was necessary to reach the equilibrium solution color under the applied electric field, because of the high viscosity of the cellulosic ChLC solutions.

Müller and Zentel [51] produced cellulosic ChLC solutions in which light modulates the color. To induce the photoresponsive color changes, they synthesized cellulose with two types of side groups: the trifluoromethyl phenyl carbamoyl group required for expressing ChLC, and the azobenzene side group that imparts photoresponsivity (Figure 11c).

Müller and Zentel [51] synthesized a cellulose derivative (azo-cellulose) by reacting 3-(trifluoromethyl)phenyl isocyanate and isocyanoazobenzene with cellulose in dimethylacetamide/LiCl solution at 80 °C for 4 d, followed by dissolution–precipitation with acetone and a methanol/water mixture. They determined the quantity of azobenzene side groups to be 5.4 wt% by UV–Vis spectroscopy. The researchers prepared ChLC solutions by dissolving azo-cellulose in diethylene glycol dimethacrylate at 44.3 wt%.

In the UV–Vis spectra of the azo-cellulose ChLC solution prior to irradiation, Müller and Zentel [51] observed the selective reflection peak at ca. 500 nm (Figure 11d). By irradiating the ChLC solution at 365 nm, the selective reflection peak shifted to 630 nm, and shifted back to ca. 500 nm by irradiation at 400 nm. This photoresponsive color change is attributable to light-induced *cis*–*trans* isomerization of the azobenzene side groups, which gives rise to a change in the cholesteric helical pitch that is attributable to the steric effect (Figure 11e). The researchers exposed the azo-cellulose ChLC solutions to irradiation for 1 h. This is indicative of a long time to reach the equilibrium of the photoresponsive color change, because of the high viscosity of the solutions.

### 3.2. Cellulosic ChLC Emulsions

A liquid-crystal emulsion is a system composed of dispersed liquid-crystal phase and a continuous liquid phase. Because the liquid-crystal phase is confined in a microscopic droplet, there are characteristic physical properties that are not observed in a bulk liquid-crystal. There have been many studies on liquid-crystal emulsions for applications to e.g., biosensors [93,94,95,96], omnidirectional laser emission [97,98,99,100], photonic pigments [101], and counterfeiting [102] This material form is expected to exhibit high macroscopic fluidity (in particular for molecular liquid-crystals, because polymer chains entangle only within each microdroplet) and high stimuli-responsivity originating from the large surface area of the microscopically dispersed liquid-crystal phase. Liquid-crystal emulsions therefore have great potential for liquid-type ChLC functional materials that overcome the drawbacks of lyotropic ChLCs mentioned in Section 3.1.

Li et al. [103] fabricated CNC-based ChLC emulsions by the microfluidic technique, and evaluated the particle size-induced topological transition of ChLC microdroplets. They also studied functionalization of the ChLC emulsions by loading nanoparticles (NPs) into the droplets.

**Figure 12:**
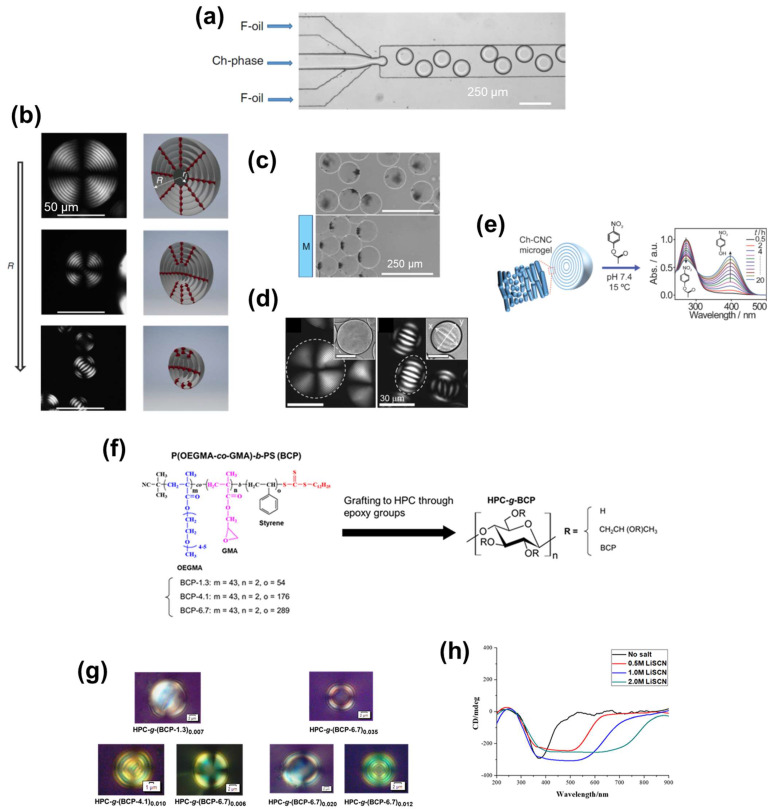
(**a**) Optical microscopy image of microfluidic emulsification of a cellulose nanocrystal (CNC) cholesteric liquid crystal (ChLC) aqueous dispersion in oil. (**b**) Polarized optical microscope (POM) images of CNC-based ChLC microdroplets with dimensions in the range of 40 ≤ *R* ≤ 115 μm, 10 ≤ *R* ≤ 40 μm, and *R* ≤ 10 μm. (**c**) Optical microscopy images of CNC-based ChLC microdroplets loaded with magnetic nanoparticles with and without an applied magnetic field ((**a**–**c**) were reproduced from [103] by permission of Nature Springer). (**d**) POM images of CNC-based ChLC microgels in water, with insets showing the corresponding bright field images. (**e**) Time dependence of the UV–Vis spectra of aqueous 4-nitrophenyl acetate mixed with CNC-based ChLC microgels ((**d**,**e**) were reproduced from [104] by permission of Wiley). (**f**) Schematic for hydroxypropyl cellulose (HPC)-*g*-block copolymer (BCP) synthesis. GMA, glycidyl methacrylate; OEGMA, oligoethylene glycol methacrylate; P(OEGMA-*co*-GMA, poly(oligoethylene glycol methacrylate-*co*-glycidyl methacrylate); PS, polystyrene. (**g**) POM images of ChLC microdroplets formed by HPC-*g*-BCPs with various PS block lengths and comparable DS_BCP_ (left three images), and with different DS_BCP_ and comparable PS block lengths (right three images). (**h**) Circular dichroism spectra of the ChLC emulsions formed by HPC-*g*-(BCP-6.7)_0.006_ with various concentrations of LiSCN ((**f**–**h**) were adapted with permission from [106], copyright (2021) American Chemical Society).

For preparation of the ChLC emulsions, Li et al. [103] injected a CNC ChLC aqueous suspension as a droplet phase into the central channel of a microfluidic device, and injected a continuous phase consisting of a fluorinated oil and a copolymer surfactant into the side channels (Figure 12a). They ranged the droplet size from the micron order to hundred-micron order with low polydispersity by changing the flow rate of the oil phase.

Li et al. [103] evaluated the effect of the droplet size on the topology of the CNC ChLC droplets by POM (Figure 12b). For the larger droplets with radius (*R*) in the range of 40 ≤ *R* ≤ 115 μm, they observed a concentric ring pattern with the Maltese cross. This optical pattern is attributable to the radial topology of the ChLC helices, where the helical axes of the ChLC are oriented perpendicular to the droplet surface (the pseudonematic planes are tangentially aligned to the droplet surface). The double distance between two adjacent concentric rings, corresponding to the cholesteric helical pitch, was 6.1 ± 0.3 μm; and the helical pitch in the droplets was independent on the droplet size. For the middle size of droplets, 10 ≤ *R* ≤ 40 μm, the researchers observed a stripe pattern at the core and the aforementioned concentric ring pattern at the periphery. This stripe pattern originates from the bipolar topology of the ChLC helices, where the helical axes of the ChLC tangentially oriented to the droplet surface. For the smaller droplets, *R* ≤ 10 μm, the stripe pattern accounted for almost all of the droplets. The researchers explained this size-dependent topological transition of the ChLC microdroplets in terms of a balance between the elastic energy of spherical packing of the ChLC helices and the surface anchoring energy of the pseudonematic planes.

To impart a range of functionalities to their CNC-based ChLC emulsions, Li et al. [103] loaded various NPs into the microdroplets. The inside of the droplets phase-separated into a CNC-rich ChLC region and an NP-rich isotropic region, whereby the ChLC structure was maintained even after loading the NPs. A ChLC emulsion with gold NP-loaded microdroplets exhibited extinction that is attributable to the surface plasmon resonance, and a ChLC emulsion with carbon dot-loaded microdroplets exhibited photoluminescence. In a CNC-based ChLC emulsion with magnetic NP-loaded microdroplets, the droplets moved in accordance with an applied magnetic field (Figure 12c).

Cho et al. [104] studied a system in which the dispersed ChLC microdroplets in CNC-based ChLC emulsion converted into microgels. They investigated the swelling behavior and microreactor characteristics of the ChLC microgels.

Cho et al. [104] prepared their ChLC emulsions by microfluidic emulsification of a CNC ChLC aqueous suspension containing a monomer (2-hydroxyethyl acrylate), crosslinker [poly(ethylene glycol) dimethacrylate], and photoinitiator in a similar manner as aforementioned. The precursor droplets exhibited a size-induced topological transition, which is consistent with the aforementioned microdroplets prepared from a simple CNC ChLC aqueous dispersion. The researchers then subjected the emulsions to UV-irradiation to transform the droplets into microgels by photopolymerization of the monomer and crosslinking the polymer chains.

Cho et al. [104] transferred the CNC-based ChLC microgels from oil to water, and examined the swelling behavior by POM (Figure 12d). The microgels obtained from the larger precursor droplets (126 μm) with radial topology exhibited isotropic swelling in water, giving rise to an increase in the particle size as well as a cholesteric helical pitch estimated from the distance between the adjacent concentric rings. However, the microgels prepared from smaller precursors (20 μm) with a bipolar topology anisotropically swelled, resulting in prolate microgels. There was preferential swelling along the direction perpendicular to the pseudonematic planes.

CNC-based ChLC microgels have the potential to act as microreactors because of the catalytic ability facilitated by the hydroxy groups that impart nucleophilicity, and anionic sulfate ester groups that impart cation-deposition properties. Cho et al. [104] examined the catalytic performance of the microgels for hydrolysis of 4-nitrophenyl acetate to 4-nitrophenolate, by monitoring the UV–Vis spectra of aqueous 4-nitrophenyl acetate mixed with CNC-based ChLC microgels. UV–Vis spectroscopy indicated that the intensity of the absorption peak at 270 nm (corresponding to 4-nitrophenyl acetate) decreased, whereas the intensity of the peak at 400 nm (corresponding to 4-nitrophenolate) increased, over the course of the reaction (Figure 12e). The researchers also produced Ag NPs in the microgels, where CNC acted as a reducing agent and an Ag^+^ ion-absorber. The CNC–Ag NP composite ChLC microgels exhibited catalytic capability for reducing 4-nitrophenol.

Wang et al. [105] produced CNC-based ChLC emulsions and ChLC microgels. They prepared the emulsions by mixing a 4 wt% CNC aqueous suspension with acrylamide, a crosslinker, and a photoinitiator; followed by emulsification in cyclohexane with the surfactant. Moreover, the researchers exposed the ChLC emulsions to UV-irradiation for photopolymerization of the acrylamide in the microdroplets to fabricate microgels incorporating the ChLC structure. Although this work did not focus on the functionalities of the ChLC emulsions and microgel suspensions, monitoring the growth of the ChLC structure in the microdroplets by POM and direct observations of the internal ChLC structure of the microgels by SEM are noteworthy.

As described previously, emulsification of CNC-based colloidal ChLC was achieved by a microfluidic technique and simple mixing with a surfactant. However, these methods are difficult to apply to cellulose derivative-based molecular ChLC because ChLC solutions of cellulose derivatives have a higher viscosity than CNC ChLC dispersions. Therefore, other approaches are required to prepare cellulose derivative-based ChLC emulsions.

Chakrabarty et al. [106] prepared cellulose derivative-based ChLC emulsions from a cellulose derivative-*g*-block copolymer (BCP) without using a microfluidic technique or adding external surfactants. In this system, the grafted BCP-side chains underwent self-assembly in a water/oil mixture because of their amphiphilicity, resulting in ChLC emulsions containing microdroplets with an inside cellulose derivative-based ChLC solution and an outside BCP layer. The researchers evaluated the relationships between the chemical structure of cellulose derivative-*g*-BCP and the physical properties of the ChLC microdroplets.

Chakrabarty et al. [106] synthesized a series of BCPs composed of hydrophilic poly(oligoethylene glycol methacrylate-*co*-glycidyl methacrylate) and hydrophobic polystyrene blocks by reversible addition–fragmentation chain transfer polymerization. They varied the ratio for the degree of polymerization of the hydrophilic and hydrophobic blocks (*x*) by changing the polystyrene block length. The researchers then grafted a series of BCPs onto HPC by ring-opening etherification involving the epoxy groups of glycidyl methacrylate and the hydroxy groups of HPC to obtain the graft copolymers HPC-*g*-(BCP-*x*)*_y_* (Figure 12f), where *y* represents the average number of grafted BCP chains per anhydroglucose unit (DS_BCP_). They prepared the ChLC emulsions by adding HPC-*g*-BCPs into a water/xylene mixture and subsequent 30-min sonication. These are water-in-oil emulsions where the HPC ChLC aqueous solution is the dispersed phase and xylene is the continuous phase. The researchers determined the quantity of water such that the HPC aqueous concentration inside the droplets was 50–70 wt%, which is required to impart a visible color to the bulk system. They formed the ChLC emulsions by sonicating the mixture for only 30 min, whereas preparation of cellulose derivative-based ChLC bulk solutions requires mixing for several days.

POM observations of the ChLC emulsions, prepared from HPC-*g*-BCPs [HPC-*g*-(BCP-1.3)_0.007_, HPC-*g*-(BCP-4.1)_0.010_, and HPC-*g*-(BCP-6.7)_0.006_] with various BCP compositions and comparable (low) DS_BCP_ values, indicate that the topological transition of the ChLC core from the radial to the bipolar occurred through an increase in the hydrophobic polystyrene length of the BCP side chains (Figure 12g). The topology of the liquid crystalline microdroplets in the emulsion was sensitive to the chemical environment at the droplet surface [93,107]. The topological transition can be interpreted as a change in the orientation state of HPC chains in response to the increase in the hydrophobicity of the droplet surface, which is imparted by the increased polystyrene block length. POM observations of ChLC emulsions, prepared from HPC-*g*-BCPs [HPC-*g*-(BCP-6.7)_0.035_, HPC-*g*-(BCP-6.7)_0.020_, and HPC-*g*-(BCP-6.7)_0.012_] with various DS_BCP_ values and an identical BCP composition, indicate that the ChLC region inside the droplets increased in accordance with increasing DS_BCP_ (Figure 12g). These POM results indicate that the mobility of the HPC molecular chains when BCP was relatively densely grafted tended to be constrained by the shell of the droplets, rendering it difficult to supply HPC segments that would form a ChLC phase to the core.

To examine the effect of adding salt to the ChLC emulsions, Chakrabarty et al. [106] prepared emulsions from HPC-*g*-(BCP-6.7)_0.006_ with LiSCN aqueous solutions at various concentrations as the water-based phase. CD spectroscopy of the ChLC emulsions with LiSCN indicated that increasing the concentration of LiSCN gave rise to a broadened CD peak of the emulsions (Figure 12h), which differed from the systematic blue-shift observed in the simple HPC aqueous solution system described in Section 3.1. This peak-broadening phenomenon is attributable to the distribution of the mobility-restricted states of the HPC chains anchored onto the BCP shell, which leads to a heterogeneous response of the cholesteric helical pitch to the salt. This unique salt-response of cellulose derivative-based ChLC emulsions can be applied to fabricate a broadband CPL filter, as revealed in Section 2.1.3.

## 4. Summary

We briefly described the fundamentals of cellulosic liquid crystals and reviewed research on fabricating cellulosic ChLC functional materials. As a concept of the review, we broadly organized such materials into the solid and liquid types, and further divided these in terms of their material forms, because the material form is foundational to the applications of the materials.

In cellulosic ChLC-based solid functional materials, films have been studied mostly because of the high film-formability of cellulosics. This material form is typically suitable for producing mechanochromic materials to visualize mechanical stimuli, such as compression and tensile. Large-scale manufacturing of touch-responsive cellulosic ChLC films using the roll-to-roll method is possible. Cellulosic ChLC films are also applicable as optical filters, and expansion of the filtering function has been accomplished by compounding cellulosic ChLCs with nematic liquid crystals and a surfactant. Cellulosic ChLC gels also are an active field of research. A typical function achieved by this material form is humidity-responsive color change. In particular, there are many reports regarding humidity-responsive hydrogels prepared from CNC-based ChLC aqueous dispersions because of the high hydrophilicity of CNC. Cellulosic ChLC gels can also exhibit a salt-responsive color change, and other responsivities can be imparted by compounding the gels with diverse synthetic polymers. Cellulosic ChLC-based mesoporous materials enable fabrication of structurally colored flexible plastics.

Regarding cellulosic ChLC-based liquid functional materials, studies on functionalization of cellulosic ChLC solutions have been limited; whereas fundamental aspects have been thoroughly investigated. This lack of functionalization research is likely because of the high viscosity of the solutions, leading to handling difficulties and slow stimuli-responses. The cellulosic ChLC emulsion is a material form that is expected to overcome these problems because of its high fluidity and the large surface area of the ChLC microdroplets. Although there are few reports on cellulosic ChLC emulsions, several interesting physical properties were found that were not observed in the bulk system. The topological transition of ChLC microdroplets in response to the surrounding chemical conditions will facilitate applications to chemical sensors. Because cellulosic ChLC microdroplets can load various NPs without disrupting the ChLC structure, researchers will develop useful functions by compounding cellulosic ChLCs with NPs. Furthermore, cellulosic ChLC microdroplets may serve as microreactors for chiral-selective reactions based on the chiral scaffold inside the droplets.

Cellulosic ChLC materials have the potential to exhibit diverse functions and possible applications, depending on their material forms, as presented in the current review. Whereas previous studies on cellulosic ChLC-based functional materials have generally focused on the coloration properties of cellulosic ChLCs, developing functions that take advantage of their chirality has been limited. Few researchers have produced functional materials, such as biomineralized films, conducting films, and mesoporous fillers, by using cellulosic ChLCs as a chiral scaffold; the relationship between the functionality of these materials and the ChLC structure remains to be clarified. To extend applications of cellulosic ChLC materials to more diverse fields of research, it may be important to facilitate such research. Another challenging subject on cellulosic ChLCs is the investigation of the relationship between the chemical structure of cellulosics and their liquid crystallinity, using precisely synthesized cellulosics. There has been no research on liquid crystallinity of cellulosics with regioselectively introduced side groups, as far as we know. In addition, the number of side groups on one repeating unit is intra- and intermolecularly random, for cellulosics used in previous research on cellulosic ChLCs. Controlling these structural parameters may enable excellent cellulosic ChLC-based functionalities. Accomplishing the subjects mentioned here will facilitate a more sustainable society by expanding the application scope of cellulosic materials.
